# Effectiveness of radiotherapy for local control in T3N0 rectal cancer managed with total mesorectal excision: a meta-analysis

**DOI:** 10.18632/oncotarget.28280

**Published:** 2022-10-08

**Authors:** Michael Jonathan Kucharczyk, Andrew Bang, Michael C. Tjong, Stefania Papatheodoru, Jesus C. Fabregas

**Affiliations:** ^1^Department of Radiation Oncology, Nova Scotia Cancer Centre, Halifax, NS B3H 1V7, Canada; ^2^Department of Radiation Oncology, Dalhousie University, Halifax, NS B3H 1V7, Canada; ^3^Department of Surgery, BC Cancer – Vancouver, Vancouver, BC V5Z 4E6, Canada; ^4^Department of Radiation Oncology, Princess Margaret Cancer Centre, Toronto, ON M5T 1W6, Canada; ^5^Department of Epidemiology, Harvard T.H. Chan School of Public Health, Boston, MA 02115, USA; ^6^Department of Medicine, University of Florida Health Cancer Center, Gainesville, FL 32610, USA

**Keywords:** radiotherapy, meta-analysis, systematic review, rectal cancer, total mesorectal excision

## Abstract

Introduction: The total mesorectal excision (TME) significantly improved rectal cancer outcomes. Radiotherapy’s benefit in T3N0 rectal cancer patients managed with TME has not been clearly demonstrated. A systematic review and meta-analysis were undertaken to determine whether radiotherapy altered the risk of locoregional recurrence (LR) in T3N0 rectal cancer patients managed with a TME.

Materials and Methods: Studies indexed on PubMed or Embase were systematically searched from inception to October 18, 2020. Preferred Reporting Items for Systematic Reviews and Meta-Analyses (PRISMA) guidelines were observed for the literature search, study screening, and data extraction; the Newcastle Ottawa Scale evaluated bias; Grades of Recommendation, Assessment, Development, and Evaluation Working Group system evaluated certainty; and all were performed independently by at least two investigators. Studies that reported LR data specific to T3N0 rectal cancer patients managed with TME, treated with and without radiotherapy, were included. Data was pooled using a random-effects model. Meta-analyses of the relative risk of local recurrence were conducted.

Results: Five retrospective cohort studies involving 932 unique patients reported LR outcomes; no prospective studies met eligibility criteria. Median follow-up ranged from 38.4–78 months. Adjuvant radiotherapy was provided in 3 studies. Chemotherapy was delivered and reported in 4 studies, providing both concurrent and adjuvant chemotherapy. A non-significant LR reduction with radiotherapy alongside TME was estimated, mean relative risk (RR) 0.63 (95% Confidence Interval 0.31–1.29; *I*^2^ = 41.8%).

Conclusions: A non-significant LR benefit with radiotherapy’s addition was estimated. Meta-analysis of exclusively retrospective cohort studies was concerning for biased results. Adequately powered randomized trials are warranted.

## INTRODUCTION

The Swedish Rectal Cancer trial established radiotherapy’s role in the management of localized rectal cancer, benefitting both locoregional recurrence (LR) and overall survival (OS) [1]. The quality of surgery has since improved dramatically with the adoption of the total mesorectal excision (TME), an en-bloc resection of the mesorectum extending to the visceral pelvic fascia [2]. TME’s use yielded results similar to what previously required both radiotherapy and less robust surgeries [3]. Once TME was combined with radiotherapy, the subsequent randomized Dutch TME trial did not demonstrate a survival benefit with radiotherapy [4, 5]. Instead, a benefit to LR was observed, though this was not statistically significant in the long-term outcomes for the Stage II subgroup (T3/4N0 participants) [6].

Given TME’s effectiveness in facilitating local control, there are reasonable arguments that the lowest risk group of the Dutch TME trial (i.e., clinically staged T3N0 rectal cancer) could forego routine radiotherapy [7]. Many established LR risk factors are absent in this population, namely a higher T-stage and lymph node involvement, though other risks may be present (ex. mucinous histology, positive surgical margins, lower tumor epicenter) [8–12]. Prospective observational studies (Mercury II, OCUM, QuickSilver) support that pre-operative magnetic resonance imaging (MRI) could allow radiotherapy to be withheld for T3N0 disease at low risk of a positive margin or nodal disease, observing positive circumferential margins in only 1.9–4.8% and 3-year LR in 1–3% of participants [13–15]. However, major guidelines suggest radiotherapy as a standard for all T3N0 rectal cancer patients [16].

Critical appraisals of radiotherapy’s benefit in the T3N0 patients are challenged by heterogeneous documentation of a high-quality TME being performed in pertinent studies. The performance of a good quality TME cannot be assumed, as it has been shown to require a formalized training program [17], a requisite for the participation in many randomized trials [18, 19]. Also, there were delays in the international adoption of TME following demonstrations of the technique’s superiority [20]. Therefore, we aimed to summarize all available evidence in a systematic review and meta-analysis to quantify the possible LR benefit from radiotherapy in T3N0 rectal cancers managed with a TME.

## RESULTS

### Screening of search results

**Figure 1:**
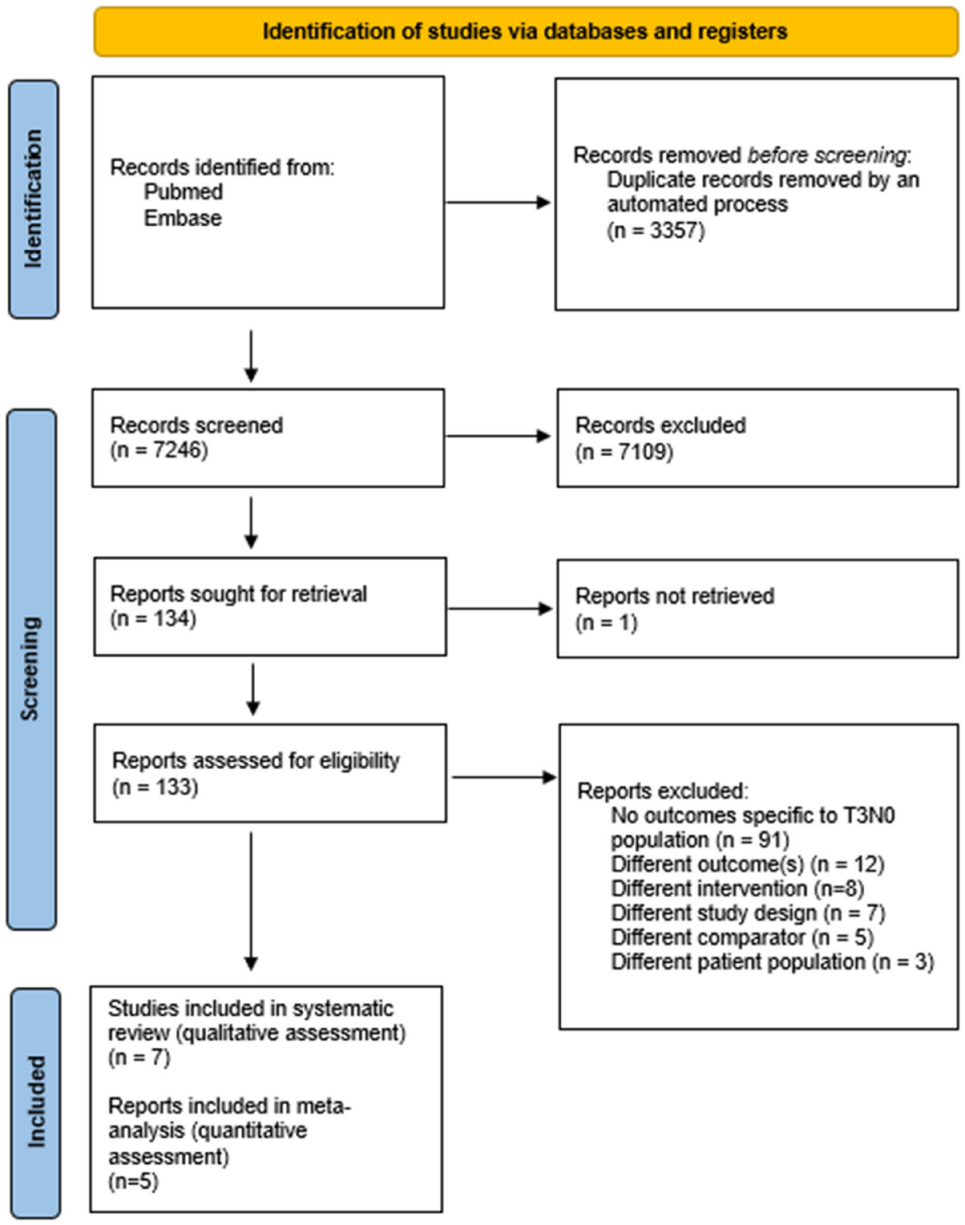
PRISMA flow chart for study selection and reporting.

The systematic search identified 7246 unique studies, of which 134 abstracts were screened as eligible and subjected to an assessment of their full-text (Figure 1). Seven unique studies were identified and subjected to assessments of quality [21–27]. Following attempted correspondence with the studies’ authors to address concerns raised when evaluating each study’s risk of bias (Supplementary Materials), five of seven studies were included for quantitative analysis [22, 25–28].

### Participant characteristics

Table 1 summarizes the characteristics of the participants of the five studies included in the meta-analysis. A summary table of the seven studies which met eligibility criteria prior to quality assessment is available in the Supplement (Supplementary Table 1).

**Table 1:** Characteristics of study participants of the five retrospective cohort studies of the meta-analysis

Trial	Country	Accrual period	Design	Participants (*n*)	Rectal cancer population	Intervention	Comparator	Median follow-up (m)	Outcome
**Delaney et al. 2002 ** **[28]**	USA	1980–2001	Retrospective Cohort	135	pT3NXM0 adenoca, <8 cm from AV	Neoadj RT + TME *40–50 Gy*	TME	41	5 yr LR
**Kim et al. 2010 ** **[22]**	South Korea	1996–2004	Retrospective Cohort	151	pT3N0 adenoca	TME + Adj RT + Adj Ctx *50.4–54 Gy*	TME + Adj Ctx	78	5 yr LR
**Peng et al. 2019 ** **[26]**	China	2005–2015	Retrospective Cohort (subgroup)	121	pT3N0M0 adenoca, <7 cm from AV, negative margins	TME + Adj CRT ± Adj Ctx *46–50 Gy*	TME + Adj Ctx	*Intervention* 56.4 *Comparator* 57.1	3 yr 5 yr LR
**Lin et al. 2019 ** **[25]**	China^**^	2010–2014	Retrospective Cohort	272	cT3N0M0 adenoca	Neoadj RT + TME + Adj Ctx *50.4 Gy*	TME ± Adj CRT^***^ ± Adj Ctx	*Intervention* 38.4 *Comparator* 46.3	2 yr LR
**Baek et al. 2020 ** **[27]**	Korea	2003–2012	Retrospective Cohort	365	pT3N0M0 adenoca, negative margins	TME + Adj CRT *43.2–60 Gy; Median 44 Gy*	TME ± Adj Ctx	71	5 yr LR

Abbreviations: adenoca: adenocarcinoma; Adj: adjuvant; AV: anal verge; CRT: concurrent chemoradiotherapy; CTX: chemotherapy; DFS: disease-free survival; TME: total mesorectal excision; LR: local or locoregional recurrence; m: months; OS: overall survival; RT: radiation therapy without concurrent chemotherapy; yr: year. ^*^TME by intent; ^**^same institution, both report use of prospective institutional database; ^***^exception, 5/75 received CRT in comparator arm.

Among the included studies, 932 participants were enrolled between 1980 and 2015. All studies involved adenocarcinoma, though cases of mucinous adenocarcinoma were documented among all 5 study populations (<10%) [21–25, 27]. Most participants, 797 (85.5%), were located in Asia [22–27]. Adjuvant chemotherapy was received by all study participants in 4 studies but was omitted in the earliest (1980) and only North American study [28]. Radiotherapy was provided adjuvantly in 3 studies [22, 26, 27] and neoadjuvantly in 2 studies [25, 28]. Median follow-up ranged from 41–78 months. The most commonly reported LR time point was at 5-years. The study that did not report 5-year LR rates had a point estimate extrapolated from figures [25]. All studies offered standard fractionated radiotherapy (1.8–2.0 Gy per fraction), with doses ranging from 40 to 60 Gy. Three studies specified their radiotherapy technique as either 3 or 4-field approaches [22, 26, 28] and two others did not specify [25, 27]. One neoadjuvant study did not control for margin status [28], two adjuvant studies included exclusively margin negative patients [26, 27], one neoadjuvant study had 5/75 participants in each arm with positive margins (with comparator patients receiving chemoradiotherapy) [25], and one adjuvant study reported 2/29 comparator participants and 7/122 intervention participants [22].

### Risk of bias

Risk of Bias was assessed in all 7 eligible studies identified by the screening process (Supplement – NOS evaluations) and is summarized in Table 2. Of note, the NOS only assesses two factors for Comparability. Tumor location and chemotherapy were deemed the two most relevant factors given that both neoadjuvant and adjuvant studies were included (biasing interpretation of margin status). Individual study concerns prompted the exclusion of two studies. GRADE assessment determined a *Low* certainty in the final result, with additional concerns for Imprecision. Expanded discussion is included in the eResults.

**Table 2:** Summary of the main outcome extracted from each study, extracting the reported 5-year local recurrence events

Study	Intervention		Comparator		Oxford quality rating	Newcastle ottawa quality assessment		
	Local recurrences	Number at risk	Local recurrences	Number at risk		Selection *(out of 4)*	Comparability *(out of 2)*	Outcomes *(out of 3)*
**Delaney et al. 2002 ** **[28]**	4 (8.3%)	48	10 (11.5%)	87	3	✯✯✯✯	✯✯	✯✯
**Kim et al. 2010 ** **[22]**	31 (25.4%)	122	6 (20.6%)	29	3	✯✯✯✯	✯	✯✯
**Lin et al. 2019 ** **[25]**	3 (2.8%)	108	2 (2.7%)	75	3	✯✯✯✯	✯✯	✯✯
**Peng et al. 2019 ** **[26]**	4 (6.9%)	58	12 (19.0%)	63	3	✯✯✯✯	✯✯	✯✯✯
**Baek et al. 2020 ** **[27]**	1 (0.1%)	143	14 (6.3%)	222	3	✯✯✯✯	✯✯	✯✯

The Oxford Centre of Evidence-based medicine Quality Rating is assigned based on study general study design, with retrospective cohort studies scored as a 3 [60]. The Newcastle Ottawa Quality Scale evaluates cohort studies based on nine separate binary classifiers, with a higher score representative of greater quality following review [23].

Publication bias was described through tests of heterogeneity, specifically an Egger test and Plot (Supplementary Figure 1) and Funnel Plot (Supplementary Figure 2). Egger’s test for small study effects did not estimate this as a significant cause of bias (*p* = 0.813). These tests did not suggest a publication bias. Due to this meta-analysis’ small sample size of studies, estimations are only provided for descriptive purposes.

### Local recurrence

**Figure 2:**
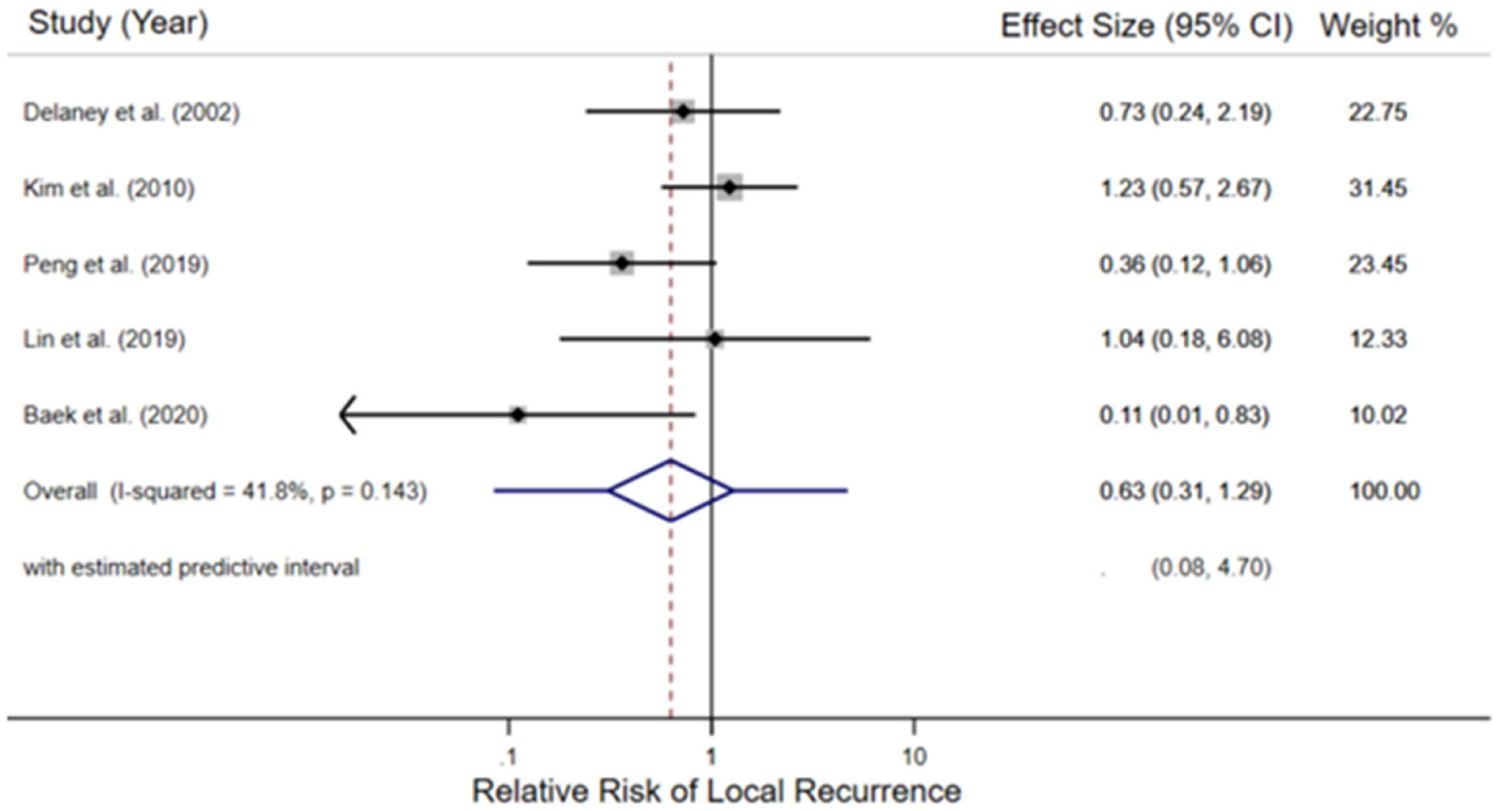
Forest plot of the relative risk of local recurrence in the included retrospective cohort studies. For each study, the black diamond indicates the point estimate, the black line the 95% confidence interval (CI), and the grey box the relative weight of the study. The hatched redline marks the point estimate of the pooled relative risk, the blue diamond portrays its 95% CI, and the blue line indicates the entire estimated predictive interval.

Of the 5 pooled trials, the random effects meta-analysis estimated the risk of LR among 955 study participants. The intervention was reported as received in 479 (radiotherapy with or without chemotherapy, concurrently and/or sequentially) and 476 were reported to receive the comparator (no radiotherapy, with or without chemotherapy). Figure 2 depicts each studies’ weighted contribution and the estimated pooled relative risk of 5-year LR of 0.63 (95% Confidence Interval (CI) 0.31–1.29, *p* = 0.143; entire predictive interval 0.08–4.70) among participants reported to have received radiotherapy compared to participants not receiving radiotherapy. The absolute number of LR events in the intervention population was 43/479 and 44/476 in the comparator. Moderate heterogeneity was observed (I^2^ = 0.41)

Sensitivity analyses established *a priori* included an influence analysis which repeated the random-effects meta-analysis, removing each study in turn, and a cumulative meta-analysis by date. The cumulative meta-analysis by date (Supplementary Figure 3) illustrates that the point estimate continuously favors radiotherapy but does not approach statistical significance. Influence analysis (Supplementary Figure 4) observed that only the removal of the Kim et al.’s works from the meta-analysis, the study where radiotherapy demonstrated the least benefit (31/122 LRs with radiotherapy versus 6/29 without), provided a pooled estimate of 5-year LR relative risk which favored radiotherapy and the 95% confidence interval did not include the null result (relative risk = 0.47; 95% CI 0.22–0.99).

## DISCUSSION

This systematic review identified unique 7 retrospective cohort studies which evaluated whether radiotherapy reduces LR in T3N0 rectal cancer patients managed with TME. Following an assessment of each studies’ risk of bias, five were included in a random-effects meta-analysis. This assessment of 932 patients observed a point estimate which signaled a benefit for radiotherapy that did not approach statistical significance. To the best of our knowledge, this is the first study to date to perform a systematic review or meta-analysis regarding the benefit of radiotherapy specific to the T3N0 rectal cancer patient population.

Existing randomized evidence does not offer a comparison of TME with or without radiotherapy specific to this group. The Dutch trial was the only trial to randomize patients treated with a TME technique by receipt of radiotherapy (versus no radiotherapy). The Dutch trial’s 492-patient clinically staged Stage II subgroup (i.e., T3N0 and T4N0) did not estimate a significant 5-year LR benefit (5.3% vs. 7.2%, *p* = 0.331) [5]. As a subgroup analysis and secondary endpoint, no strong conclusions should be drawn from this finding due to a lack of appropriate power to detect a possible effect. There are additional issues limiting indirect comparisons of the Dutch’s population to this meta-analysis – the Dutch’s participants were not allowed chemotherapy while adjuvant chemotherapy was either provided or offered for 909/1044 (87%) of this meta-analysis’ participants. Thus, it is unclear if all stage II patients are at sufficient risk of locoregional dissemination to merit radiotherapy’s modest absolute benefit and known adverse effects [29–34].

Two SEER-based population-level studies attempted to clarify this question for T3N0 patients [35, 36]. The first explored radiotherapy’s possible benefit in 4724 rectal cancer patients, observing a potentially statistically significant benefit in cancer-specific survival among patients receiving adjuvant radiotherapy compared to those that did not (HR = 0.69, 95% CI = 0.58–0.82, *p* < 0.001). Though this was not observed in those receiving neoadjuvant radiotherapy (HR = 0.86, 95% CI = 0.72–1.04, *p* = 0.13) [35]. A second study only observed a benefit with radiotherapy in those with high-risk disease, defined as an age ≥70 or the combination of grade III/IV disease with less than 12 nodes resected [36]. Issues which affect population-based studies may be prevalent in these studies, including the inability to control for all relevant confounders or selection bias. Moreover, they did not ascertain whether patients underwent TME.

Prospective evidence includes three observational series which used MRI to direct management of early rectal cancer patients, including cT3N0 disease [13–15]. As all reported acceptably low LR rates in patients foregoing radiotherapy, if deemed low-risk by MRI, they could be interpreted as suggested scenarios where radiotherapy can selectively omitted. Results specific to T3N0 patients were not uniformly reported.

Among randomized evidence, the maturing PROSPECT compares neoadjuvant approaches for cT2N1 and cT3N0-1 disease, chemoradiotherapy versus multiagent chemotherapy with selective chemoradiotherapy, [37]. Elsewise, the existing generation of randomized radiotherapy evidence is either exploring intensification via a total neoadjuvant approach [38–40] and/or de-intensification by sparing patients a TME [41, 42]. Studies investigating a total neoadjuvant approach did not randomize radiotherapy’s provision, barring any further signal to assist with this meta-analysis’ question.

While the best available randomized data, upcoming evidence, nor this meta-analysis sufficiently support the consideration of radiotherapy for all T3N0 disease that will receive a good quality TME, prospective observational evidence has suggested low-risk patients can be selected to forego radiotherapy while high-risk patients have a LR rate which could benefit from radiotherapy. A strength of this meta-analysis is identifying all available comparable studies that specifically reported on T3N0 rectal cancer. Modest support for this result were moderately consistent results (I^2^ = 0.41) and acceptable risks of bias for cohort studies (as per the NOS scores). Given that there is nodal positivity observed in approximately 20% of clinically staged T3N0 cases [43], it was reassuring to observe that radiotherapy did not clearly have a benefit in the two populations managed neoadjuvantly [26, 28], relative to the three studies where clinical decision making had the benefit of definitive surgical pathology [22, 25, 27].

There are significant limitations to this meta-analysis. Foremost is the quantity and quality of the data – ultimately there are only 932 participants among 5 retrospective cohort studies. The NOS evaluation also does not adequately address concerns of Comparability, as there is an increased risk of LR with a positive margin or adjuvant radiotherapy [44]. As per our GRADE evaluation, this would limit the certainty of this meta-analysis’ estimated point estimate and range of error to Low. Further concerns relate to generalizability regarding the meta-analysis population’s ethnicity, receipt of chemotherapy, presence of a threatened margin, mixed inclusion of adjuvant and neoadjuvant therapies, and the broad confidence interval.

Though all but one study reported margin status, neither neoadjuvant study directed management based on a threatened margin and two of three adjuvant studies exclusively treated margin negative patients. The exception is one study offered adjuvant chemoradiotherapy to comparator participants with positive margins [25]. As randomized evidence supports that neoadjuvant radiotherapy downsizes disease and secondary to the assessment of margin status in this meta-analysis’ studies, our results should not be generalized to patients where surgical margins are threatened. [45–47].

There may also be regional generalizability concerns as four of the five studies were performed in continental Asia [22, 25–27]. There is limited evidence to provide meaningful guidance as to whether Asian American populations have different outcomes from other Americans nor any other studies that have compared localized rectal cancer outcomes in continental Asia to elsewhere [48, 49]. Unlike the single North American study [21, 28], the participants in the Asian studies also received either concurrent or multi-agent chemotherapy – further miring comparability.

Though it was reassuring to see similar outcomes being observed in the two included studies which exclusively included participants with low rectal cancers, this benefit was not observed homogeneously throughout the analyzed studies [21, 26]. In contrast, Baek et al’s. study of Korean T3N0 patients noted that either a low rectal cancer (<5 cm from the anal verge) or a close surgical margin, signaled for a possible benefit with concurrent chemoradiotherapy [27].

Translation of this data is further limited by the diverse integration of adjuvant chemotherapy evidence in rectal cancer. Generalizability issues include that randomized colorectal cancer chemotherapy trials often excluded rectal cancer patients to avoid any confounding toxicity signals from radiotherapy [50], studies exclusive to rectal cancer did not clearly control for TME, outcomes specific to the T3N0 population were not reported, nor was LR reported [51, 52]. The interpretation of the randomized evidence has thus prompted varied provision of adjuvant chemotherapy for intermediate risk rectal cancer – including T3N0 patients [53–55]. Given that adjuvant chemotherapy was offered routinely in 5 out of 6 of this meta-analysis’ studies, it would be difficult to apply this study’s results to patients not receiving systemic therapy.

A modestly sized randomized control trial would have reasonable power to resolve this question. Allowing for a one-sided evaluation (α = 0.05; β = 0.8) with this meta-analysis’ point estimate for benefit (HR = 0.63), a one-to-one randomized study would only need to treat 120 participants. This increases to 139 participants if the Dutch TME’s Stage II subgroup data was used (HR = 0.71). To address other confounders, controlling for chemotherapy use and only including patients with unthreatened margins would seem prudent. Another practical approach, albeit with lower methodological rigor, would be an ad-hoc analysis of radiotherapy’s benefit among the Dutch TME’s T3N0 participants.

With low certainty, this meta-analysis observed a non-significant benefit with radiotherapy to 5-year LR rates among T3N0 rectal cancer patients that received a TME. Until a pragmatically sized randomized control trial is completed, our research adds a layer of data to facilitate informed and personalized treatment decisions for T3N0 rectal cancer patients, albeit with potential significant bias from solely relying on retrospective cohort studies.

## MATERIALS AND METHODS

Systematic review and meta-analysis were performed and reported as per the Preferred Reporting Items for Systematic Reviews and Meta-Analyses (PRISMA) guidelines [56]. A study protocol was registered to the PROSPERO data base (CRD42020216058). Meta-analysis of Observational Studies in Epidemiology and PRISMA checklists were reported [57]. Four oncologists (AB, JF, MJK, MT) performed title and abstract screening, assessment of eligibility criteria, data extraction, and assessments of bias. Two authors were involved in either reviewing or screening any given item. A third author resolved inconsistencies, except in the case of data extraction. All authors reviewed and confirmed correct data extraction.

### Search strategy and selection criteria

A search strategy was developed in collaboration with professional librarian services (Countway Library, Boston, MA, USA). The search was restricted to English language literature reported in PubMed and EMBASE from inception to October 18, 2020. Four search hedges were utilized requiring a description of radiotherapy, rectal cancer, an interventional study, and a surgical resection. The supplement details the full search strategy (Supplementary Materials).

Inclusion criteria screening and full text assessment was performed via the Covidence platform (Melbourne, Australia). Full texts were then assessed for eligibility criteria. Included studies had their reference lists considered for potential studies that would meet inclusion criteria.

### Eligibility criteria

Studies were considered eligible if they published local or LR rates in a T3N0M0 rectal cancer population where all participants were explicitly stated to have had a TME, if they had an intervention arm which received radiotherapy, and if they had a comparator arm which did not receive radiotherapy. Studies which published a Duke’s staging equivalent to T3N0M0 were eligible. Studies could clinically and/or pathologically stage their patients to qualify for the study. Radiotherapy of any dose and fractionation combination, with or without concurrent chemotherapy, qualified as receiving radiotherapy.

### Data extraction

The following data was extracted into a dedicated database: study characteristics, baseline participant demographics, accrual dates, treatment modalities, confirmation that TME was performed, follow-up duration, LR, and OS rates. Outcome event rates were extracted for all reported time points. Inconsistencies were resolved by discussion.

### Outcomes

The primary outcome of this meta-analysis was LR, defined as a recurrence in the pelvis, with or without other distant disease. Thus, LR was an aggregate outcome for locoregional control when locoregional control was also reported. *A priori*, it was decided to extract all reported LR rates then present the most commonly reported time-point among the eligible studies as the LR time-point. This reduced the risks associated with interpolating data, which could have occurred if we had specified a time-point that was ultimately not commonly reported.

### Assessment of quality (certainty and risk of bias)

The Newcastle Ottawa Scale (NOS) was used to systematically evaluate each eligible study’s risk of bias [58]. The NOS provided a framework to assess cohort studies, evaluating their selection of the exposed and non-exposed, ascertainment of the exposure, comparability of the cohorts based on methodological considerations, assessment of the outcome, duration of follow-up, and the adequacy of follow-up. When assessing comparability, the two most relevant factors must be selected by investigators *a priori*. Studies were investigated if they controlled for either tumor location within the rectum or receipt of multiagent chemotherapy. The Grades of Recommendation, Assessment, Development, and Evaluation Working Group system (GRADE) was used to grade the certainty of the result based on the evidence’s risk of bias, inconsistency, indirectness, imprecision, publication bias, large effects, dose response, and opposing plausible residual bias and confounding [59]. Two authors (JF, MJK) performed GRADE assessment was; discrepant opinions were resolved through discussion. Certainty and risk of bias assessment required review of each study’s full text. Following quality assessment, attempts to contact corresponding author(s) for each included study of the meta-analysis was attempted to resolve any identified uncertainties.

### Data synthesis and analysis

For each trial, if they were not reported, 5-year LR rates were calculated. These values were used as inputs to calculate weight pooled treatment effects and a 95% confidence interval (CI) using a random-effects meta-analysis. Variance was estimated by using the DerSimonian and Laird approach.

*I^2^* statistics and forest plots were used to assess for heterogeneity across studies. Forest plots facilitated an influence analysis where each study was removed sequential and a cumulative random-effects meta-analysis by the date of publication. *Ad hoc* sensitivity analyses included repeating the influence analysis without a specific study that had questionable adherence to implementing TME surgery [21]. Publication bias was evaluated by Egger and Funnel plots.

## SUPPLEMENTARY MATERIALS







## Abbreviations

CI: Confidence Interval
GRADE: Grades of Recommendation, Assessment, Development, and Evaluation Working Group system
Gy: Gray
LR: Local Recurrence
OS: Overall Survival
MRI: Magnetic Resonance Imaging
NOS: Newcastle Ottawa Scale
PRISMA: Preferred Reporting Items for Systematic Reviews and Meta-Analyses
TME: Total Mesorectal Excision
RR: Relative Risk
