# Addendum: Effectiveness of radiotherapy for local control in T3N0 rectal cancer managed with total mesorectal excision: a meta-analysis

**DOI:** 10.18632/oncotarget.28484

**Published:** 2023-08-30

**Authors:** Michael Jonathan Kucharczyk, Andrew Bang, Michael C. Tjong, Stefania Papatheodorou, Jesus C. Fabregas

**Affiliations:** ^1^Department of Radiation Oncology, Nova Scotia Cancer Centre, Halifax, NS B3H 1V7, Canada; ^2^Department of Radiation Oncology, Dalhousie University, Halifax, NS B3H 1V7, Canada; ^3^Department of Surgery, BC Cancer – Vancouver, Vancouver, BC V5Z 4E6, Canada; ^4^Department of Radiation Oncology, Princess Margaret Cancer Centre, Toronto, ON M5T 1W6, Canada; ^5^Department of Epidemiology, Harvard T.H. Chan School of Public Health, Boston, MA 02115, USA; ^6^Department of Medicine, University of Florida Health Cancer Center, Gainesville, FL 32610, USA

**This article has an addendum:** No external funding was obtained.

Original article: Oncotarget. 2022; 13:1109–1119. 1109-1119. https://doi.org/10.18632/oncotarget.28280

