# Supplementary material for: Effectiveness of radiotherapy for local control in T3N0 rectal cancer managed with total mesorectal excision: a meta-analysis
Source: Oncotarget. 2022 Oct 8;13:1109–19. doi: 10.18632/oncotarget.28280 (PMC9564357; doi:10.18632/oncotarget.28280)
Supplement: Supplementary file 1 [file oncotarget-13-28280-s001.pdf]

Effectiveness of radiotherapy for local control in T3N0 rectal cancer managed with total mesorectal excision: A meta-analysis

SUPPLEMENTARY MATERIALS

Search strategy

Each search contained four hedges, describing:

1. Radiotherapy (Keywords: *radiotherapy, radiation, irradiation, chemoradiotherapy*).
2. Rectal cancer (Keywords: *rectal* or *rectum* and any of *cancer, tumour, tumor, carcinoma, adenocarcinoma, neoplasm*).
3. Interventional studies (Keywords: *randomized controlled trial, controlled clinical trial, cohort study, case-control study, comparative study, multivariate*).
4. Surgical resection (Keywords: *rectum resection, surgery, surgical, resection, total mesorectal excision, TME*).

Embase

('radiotherapy'/exp OR radiotherap*:ab,ti OR 'radiation':ab,ti OR 'irradiat*':ab,ti OR 'chemoradiotherap*':ab,ti OR 'radiochemotherap*':ab,ti)

AND

('rectal neoplasms'/exp OR ((rectum OR rectal) adj3 (cancer* OR tumor* OR tumour* OR carcinoma* OR adenocarcinoma* OR neoplasm*)):ab,ti)

AND

('randomized controlled trial'/it OR 'controlled clinical trial'/it OR 'random*':ab,ti OR 'placebo':ab,ti OR 'randomly':ab,ti OR 'trial':ab,ti OR 'cohort studies'/exp OR 'case-control studies'/exp OR 'comparative study'/it OR 'risk factors'/exp OR 'cohort':ab,ti OR 'compared':ab,ti OR 'groups':ab,ti OR 'case control':ab,ti OR 'multivariate':ab,ti)

AND

('rectum resection'/exp OR surgery:ti,ab OR surgeries:ti,ab OR surgical:ti,ab OR resection*:ti,ab OR 'total mesorectal excision':ti,ab OR tme:ti,ab)

AND

#1 AND #2 AND #3 AND #4 AND #5 AND [english]/lim NOT 'conference abstract'/it

Pubmed -

("Radiotherapy"[mesh:noexp] OR "radiotherapy"[sh] OR radiation[tiab] OR radiotherap*[tiab] OR irradiat*[tiab] OR chemoradiotherap*[tiab] or radiochemotherap*[tiab])

AND

("Rectal Neoplasms"[Mesh:noexp] OR rectal cancer*[tiab] OR rectal tumor*[tiab] OR rectal tumour*[tiab] OR rectal neoplasm*[tiab] OR cancer of the rectum[tiab])

AND

("randomized controlled trial"[pt] OR "controlled clinical trial"[pt] OR random*[tiab] OR placebo[tiab] OR randomly[tiab] OR trial[tiab] OR groups[tiab] OR "cohort studies"[mesh] OR "case-control studies"[mesh] OR "comparative study"[pt] OR "risk factors"[mesh] OR cohort[tiab] OR compared[tiab] OR groups[tiab] OR case control[tiab] OR multivariate[tiab])

AND

("Proctectomy"[Mesh] OR "Rectum/surgery"[mesh] OR "Rectal Neoplasms/surgery"[Mesh] OR surgery[tiab] OR surgeries[tiab] OR surgical[tiab] OR resection*[tiab] OR "total mesorectal excision"[tiab] OR tme[tiab])

Results – assessment of quality

Risk of bias

The Newcastle Ottawa Scale assessment for Risk of bias prompted concerns for exclusion among three studies. Following correspondence with the studies' authors and group discussion on the provided materials, ultimately five of seven studies were deemed sufficient for inclusion. More specifically, two studies originated from the same prospective database, with 2 years of overlap in their study accrual [23, 25]. Correspondence confirmed that results could not be obtained without allowing for some population overlap and both studies were felt to be of a similar risk of bias (as per the Newcastle Ottawa Scale assessment), thus the study with larger sample size was selected to be kept to maximize the power of the meta-analysis [25]. A separate study acknowledged that while TME was the surgical intervention of choice by regional guidelines, regional data suggested that study participants would have had a reasonable possibly of *not* receiving a TME [16, 21]. Following correspondence, the study was deemed to not meet inclusion criteria as results specific to a population which likely received TME could not be ascertained. The corresponding author of one study clarified that all participants received six cycles of adjuvant chemotherapy and all radiotherapy included concurrent chemotherapy [22].

One study was granted an exception for inclusion. In this study of neoadjuvant concurrent chemoradiotherapy, adjuvant chemoradiotherapy was offered to 5/75 comparator participants if they had positive margins or pT4 disease [25]. The reviewers granted this study an exception as these 5 participants would have been deemed comparable to the intervention arm prior to surgery (i.e., *a priori*).

Among the five trials included in the meta-analysis, the risk of bias was considered low after acknowledging the risk of bias intrinsic to retrospective cohort studies. Selection of patients were found to be representative within the predetermined eligibility criteria of T3N0 rectal cancers and both control and intervention cohorts were found to be drawn from the same community in all 5 studies. Additionally, data was ascertained through a secure record with objective inputs which could be compared between studies. Amongst the categories of bias, the quality of comparability and outcomes were at greatest risk. The heterogeneity of patient/treatment characteristics between studies affected the comparability, specifically in the domains of tumor location within the rectum (upper, middle, lower) and use of chemotherapy (neoadjuvant and/or adjuvant). A thorough description and determination of the nature of subjects lost to follow-up was not reported.

Assessment of certainty

The GRADE assessment of the overall certainty of the body of evidence as it related to LR as an outcome was deemed Low, consistent with observational studies of an acceptable quality. Review of the GRADE criteria, with assistance of the Newcastle Ottawa Scale, noted that Indirectness was reasonable as all studies focused on the T3N0 rectal cancer subpopulation. However, the small number of events and broad confidence intervals which could not suggest a benefit or harm raised concerns for Imprecision. Evaluations of Inconsistency, Publication Bias, Large Effects, Dose Response, and Opposing plausible residual bias & confounding did not identify sufficient concerns or strengths to suggest further modifying the certainty of the evidence beyond Low (the default assessment for observational studies of an acceptable quality).

Supplementary Material 1: MOOSE checklist. See Supplementary Material 1

Supplementary Material 2: Newcastle Ottawa Scale assessments. See Supplementary Material 2

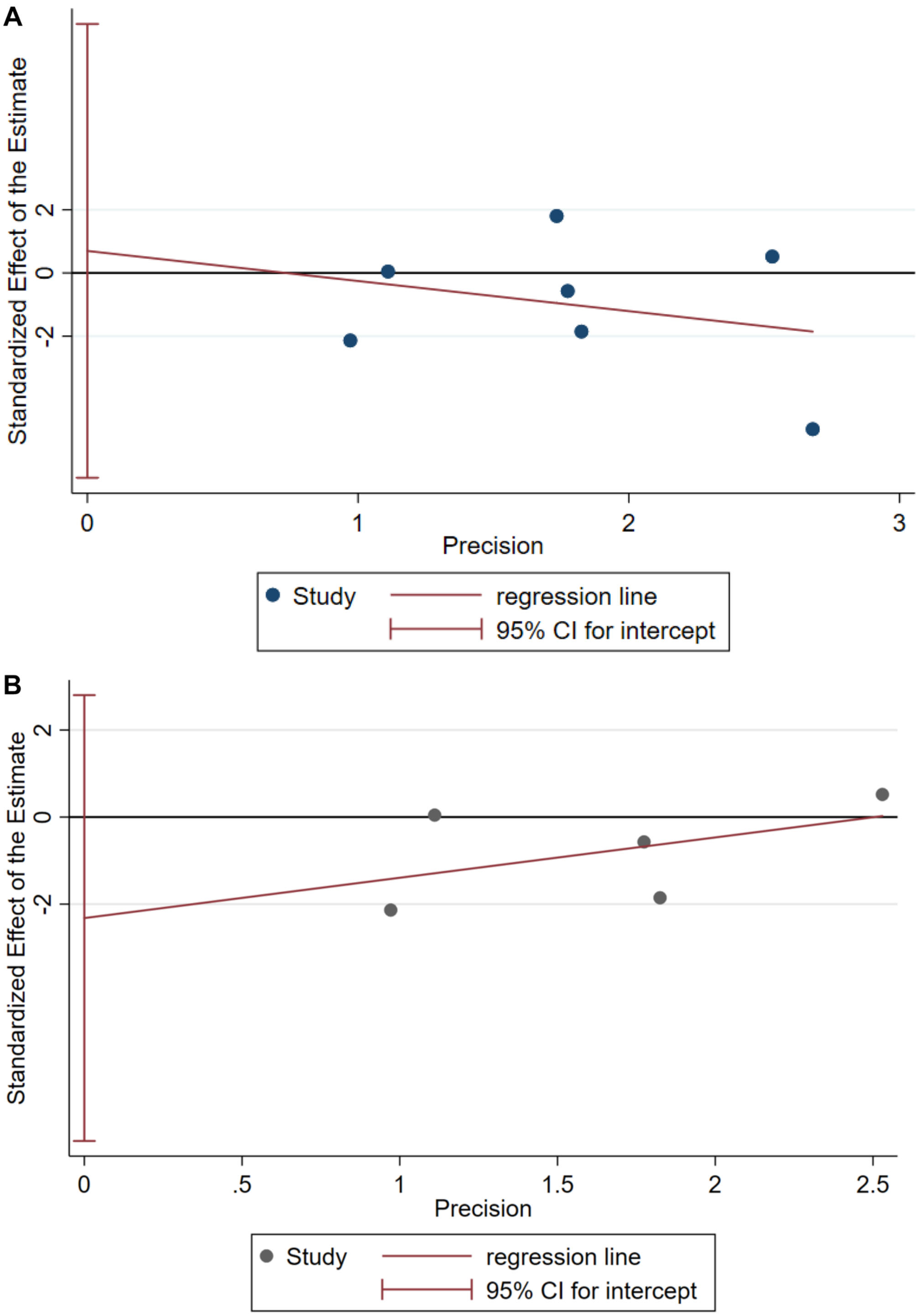

Supplementary Figure 1: Egger plot for publication bias. (**A**) Funnel plot A is for all 7 studies subjected to qualitative analysis. (**B**) Funnel plot B is for the 7 studies subjected to the quantitative analysis.

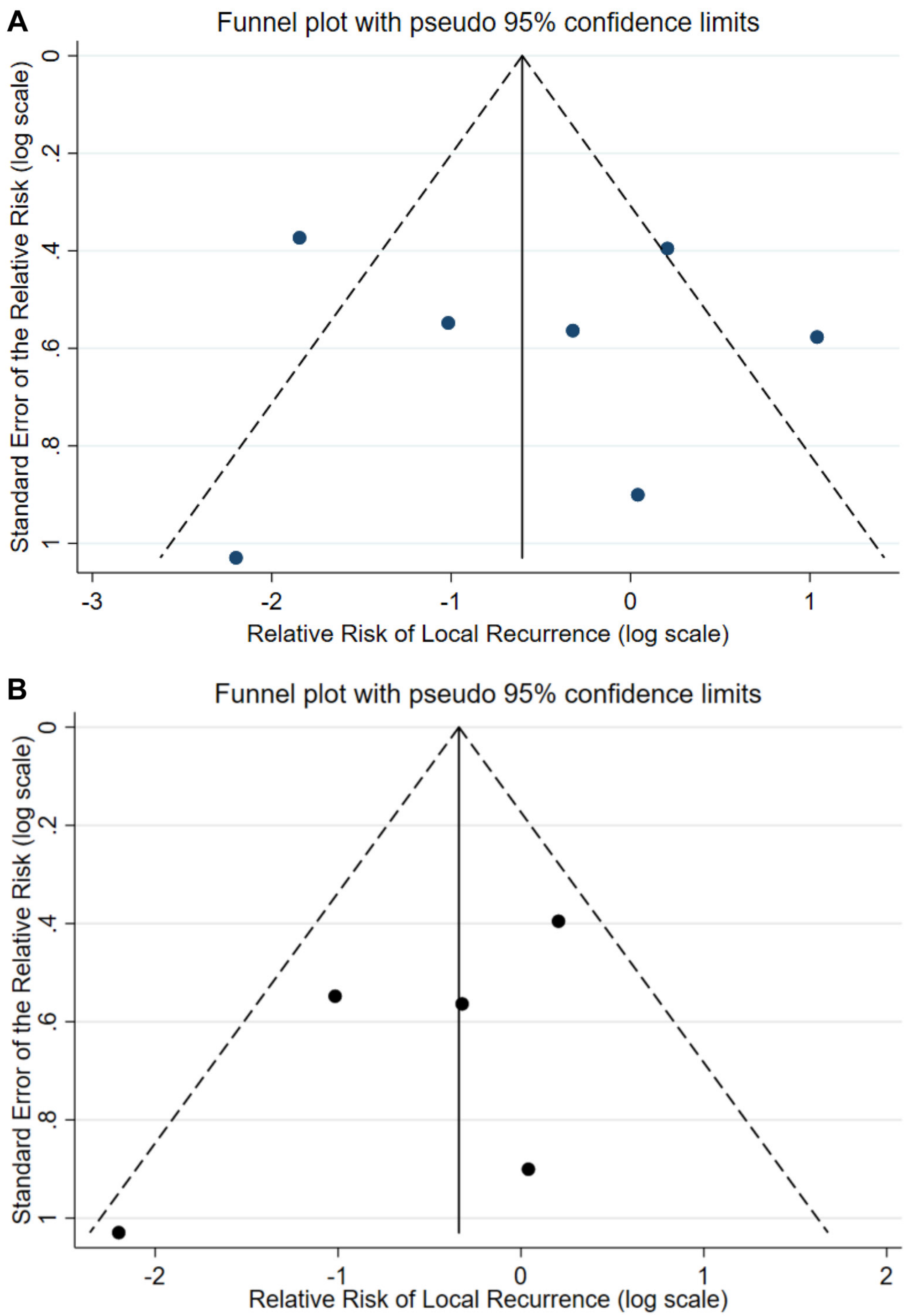

Supplementary Figure 2: Funnel plot for publication bias. (**A**) Funnel plot A is for all 7 studies subjected to qualitative analysis. (**B**) Funnel plot B is for the 5 studies subjected to the quantitative analysis.

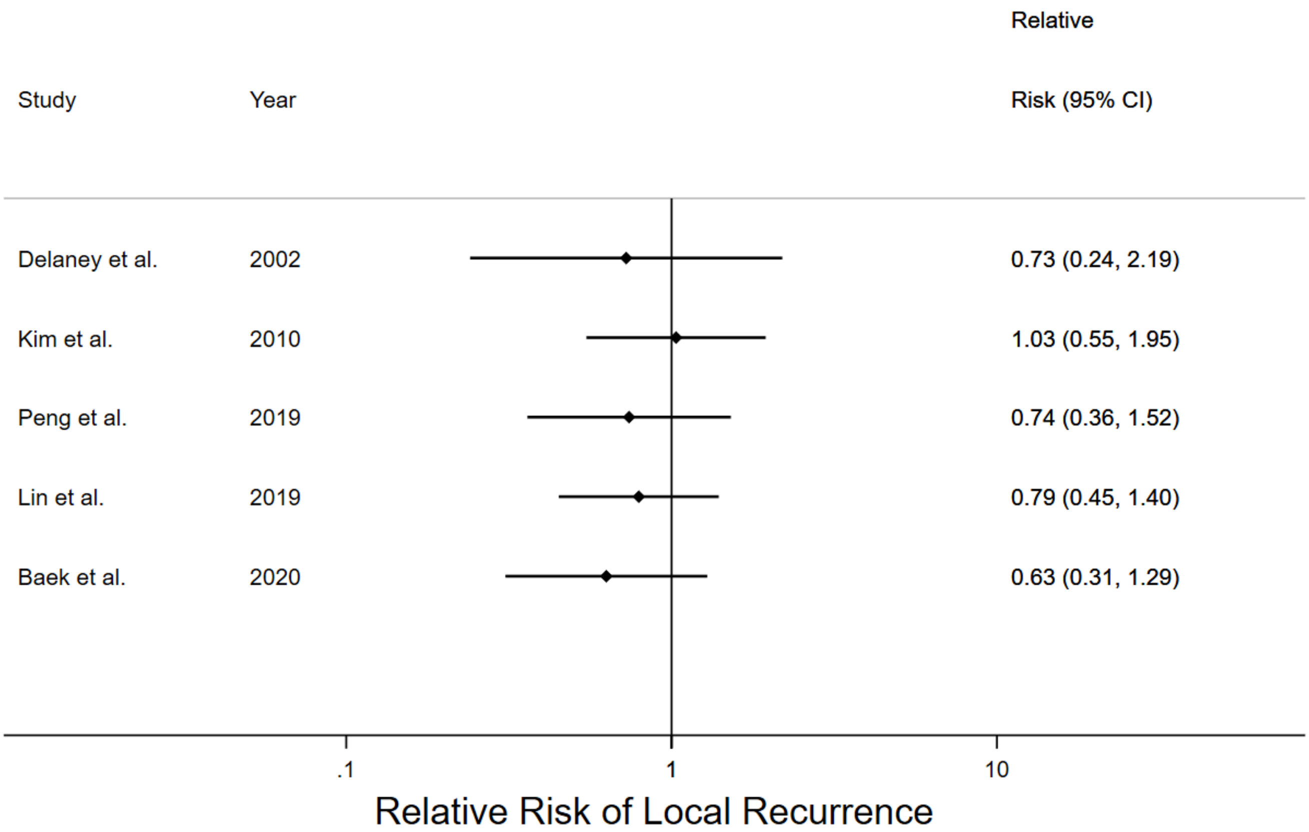

Supplementary Figure 3: Cumulative meta-analysis by date of publication.

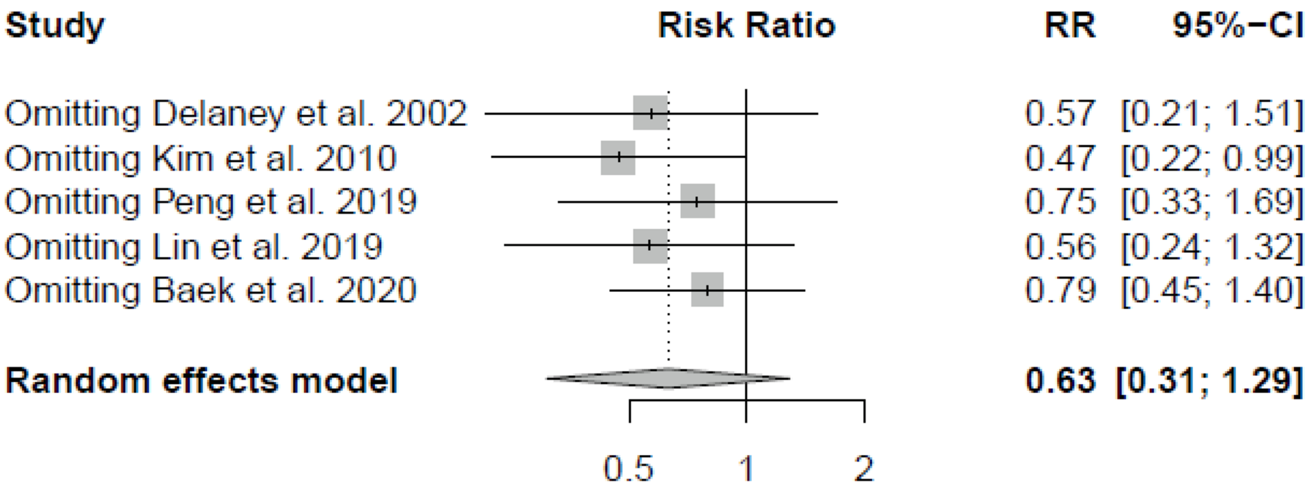

Supplementary Figure 4: Influence analysis.

Supplementary Table 1: Characteristics of included studies and two additional cohort studies dropped secondary to concerns for bias

Trial	Country	Accrual period	Design	Participants (*n*)	Rectal cancer population	Intervention	Comparator	Median Follow-up (m)	Outcomes
**Delaney et al. 2002 [28]**	USA	1980–2001	Retrospective Cohort	135	pT3NXM0 adenoca, <8 cm from AV	Neoadj RT + TME 40–50 Gy	TME	41	5yr LR
**Kim et al. 2010 [22]**	South Korea	1996–2004	Retrospective Cohort	151	pT3N0 adenoca	TME + Adj RT + Adj Ctx 50.4–54 Gy	TME + Adj Ctx	78	5yr LR
**Kennecke et al. 2012 [21]**	Canada	2000–2004	Retrospective Cohort	307	pT3N0M0 adenoca or ypT3N0M0 adenoca	Surgery* + Adj CRT ± Adj Ctx 25 Gy in 5 fractions or Neoadj RT + Surgery* ± Adj Ctx 45 Gy ± 9 Gy boost	Surgery*	62	5yr LR
**Wu et al. 2014 [23]**	China**	2003–2011	Retrospective Cohort	141	pT3N0M0 adenoca	TME + Adj CRT + Adj Ctx	TME + Adj Ctx	44	5yr LR
**Lin et al. 2019 [25]**	China**	2010–2014	Retrospective Cohort	272	cT3N0M0 adenoca	Neoadj RT + TME + Adj Ctx 50.4 Gy	TME ± Adj CRT*** ± Adj Ctx	Intervention 38.4 Comparator 46.3	2yr LR
**Peng et al. 2019 [26]**	China	2005–2015	Retrospective Cohort (subgroup)	121	pT3N0M0 adenoca, <7 cm from AV, negative margins	TME + Adj CRT ± Adj Ctx 46–50 Gy	TME + Adj Ctx	Intervention 56.4 Comparator 57.1	3yr 5yr LR
**Baek et al. 2020 [27]**	Korea	2003–2012	Retrospective Cohort	365	pT3N0M0 adenoca, negative margins	TME + Adj CRT 43.2–60 Gy; Median 44 Gy	TME ± Adj Ctx	71	5yr LR

Abbreviations: adenoca: adenocarcinoma; Adj: adjuvant; AV: anal verge; CRT: concurrent chemoradiotherapy; CTX: chemotherapy; DFS: disease-free survival; TME: total mesorectal excision; LR: local or locoregional recurrence; m: months; OS: overall survival; RT: radiation therapy without concurrent chemotherapy; yr: year. *TME by intent; **same institution, both report use of prospective institutional database; ***exception, 5/75 received CRT in comparator arm.
