# Supplementary material for: Effectiveness of radiotherapy for local control in T3N0 rectal cancer managed with total mesorectal excision: a meta-analysis
Source: Oncotarget. 2022 Oct 8;13:1109–19. doi: 10.18632/oncotarget.28280 (PMC9564357; doi:10.18632/oncotarget.28280)
Supplement: Supplementary file 3 [file oncotarget-13-28280-s003.pdf]

Supplementary Material 2: Newcastle Ottawa Scale assessments

Study Lead Author	Selection (X/4)	Comparability (X/2)	Outcomes (X/3)	*Total (X/9)*
Delaney	#REF!	#REF!	#REF!	*#REF!*
Kim	#REF!	#REF!	#REF!	*#REF!*
Kennecke	#REF!	#REF!	#REF!	*#REF!*
Wu	#REF!	#REF!	#REF!	*#REF!*
Lin	#REF!	#REF!	#REF!	*#REF!*
Peng	#REF!	#REF!	#REF!	*#REF!*
Baek	#REF!	#REF!	#REF!	*#REF!*

RESULTS BY STUDY

First Author: Delaney **Total** 8

SELECTION

Total Selection Score: 4 Out of 4

Representativeness of the Exposed Cohort

Consensus Score: 1 Report as 0 or 1

Reviewer 1 *Reviewer 2* *Conflicts*

AB	JF	MK	Value	Description
		[redacted]	*STAR*	Truly representative of T3N0 rectal cancer patients in the community
X	X		*STAR*	Somewhat representative of T3N0 rectal cancer patients in the community
			-	Not well representing T3N0 rectal cancer patients in the community
			-	No description of the derivation of the cohort

Open Comments: AB - Examined "low" rectal cancers from 5 cm or closer to anal verge
JF: Low rectal cancer, therefore, somewhat representative.

Selection of the Non-Exposed Cohort

Consensus Score: 1 Report as 0 or 1

Reviewer 1 *Reviewer 2* *Conflicts*

AB	JF	MK	Value	Description
	X	X	*STAR*	Drawn from the same community as the exposed cohort
X			-	Drawn from a different community than the exposed cohort
			-	No description of how the non-exposed cohort was derived

Open Comments: AB - Difference seen in age, sex between cohorts
JF: Age, Sex not that much different. P value not signifcant. Samples drawn from same retrospective database.
MK - it is not a comparison of the study population, asking if same community; since one hospital, this is likely

Ascertainment of Exposure

Consensus Score: 1 Report as 0 or 1

Reviewer 1 *Reviewer 2* *Conflicts*

AB	JF	MK	Value	Description
X	X	[redacted]	*STAR*	From a secure record (ex. Medical records)
			STAR	From a structured interview of a participant
			-	Written self-report by the participant
			-	No description of how the exposure was ascertained

Open Comments:

Demonstration that local recurrence had not already occurred at the start of the study

Consensus Score: 1 Report as 0 or 1

Reviewer 1 *Reviewer 2* *Conflicts*

AB	JF	MK	Value	Description
X	X	[redacted]	*STAR*	Yes
			-	No

Open Comments:

COMPARABILITY

Comparability of cohorts on the basis of the design or analysis

Total Comparability Score: 2 Out of **2** (note that the study can store two points in this single category)

Reviewer 1 *Reviewer 2* *Conflicts*

AB	JF	MK	Value	Description
X	X	[redacted]	*STAR*	Study controls for tumour location
X	X		*STAR*	Study controls for chemotherapy (multi-agent; neoadjuvant and/or adjuvant)

Open Comments: AB - Patients were all "low", patients with chemo were excluded (outside of PRT)
JF: Low rectal tumors, no chemotherapy.

OUTCOME

Total Selection Score: 2 Out of 3

Assessment of Outcome

Consensus Score: 1 Report as 0 or 1

Reviewer 1 *Reviewer 2* *Conflicts*

AB	JF	MK	Value	Description
X	X	[redacted]	*STAR*	Independent blind assessment (ex. Linked to medical records)
			STAR	Record linkage (ex. Automated database extraction, like billing codes)
			-	Self-reported by participant (i.e. no reference to the medical record)
			-	No description

Open Comments: AB - prostpectively maintained database (MJK - I deleted your weaker vote, since you scored the highest one already)
JF: confirmation of outcome by eamination of records.

Was follow-up long-enough for outcomes to occur

Consensus Score: 1 Report as 0 or 1

Reviewer 1 *Reviewer 2* *Conflicts*

AB	JF	MK	Value	Description
X	X	[redacted]	*STAR*	Yes (Median FU: 3 years)
			-	No

Open Comments: AB - 41 months

Adequacy of Follow-up

Consensus Score: 0 Report as 0 or 1

Reviewer 1 *Reviewer 2* *Conflicts*

AB	JF	MK	Value	Description
		[redacted]	*STAR*	Complete follow-up - all subjects accounted for
			STAR	Subjects lost to follow-up unlikely to introduce bias - small number lost (85% have adeq FU)
			-	Follow-up rate was under 85% and no description for those lost to follow-up
X	X		-	No statement

Open Comments: AB - this is tough as there's no statement, but hard to ascertain
JF: Unclear adequacy of follow up.

First Author: Kim **Total** 7

SELECTION

Total Selection Score: 4 Out of 4

Representativeness of the Exposed Cohort

Consensus Score: 1 Report as 0 or 1

Reviewer 1 *Reviewer 2* *Conflicts*

AB	MK	MT	Value	Description
X		[redacted]	*STAR*	Truly representative of T3N0 rectal cancer patients in the community
	X		*STAR*	Somewhat representative of T3N0 rectal cancer patients in the community
			-	Not well representing T3N0 rectal cancer patients in the community
			-	No description of the derivation of the cohort

Open Comments: MJK - less comorbid in this study, higher than expected amount of positive margins; Likely exclusively asian population
AB - I think the postiivity rate of 6,7% is within reason. Comorbidities are also reasonable (not a lung cancer population)

Selection of the Non-Exposed Cohort

Consensus Score: 1 Report as 0 or 1

Reviewer 1 *Reviewer 2* *Conflicts*

AB	MK	MT	Value	Description
X	X	[redacted]	STAR	Drawn from the same community as the exposed cohort
		[redacted]	-	Drawn from a different community than the exposed cohort
		[redacted]	-	No description of how the non-exposed cohort was derived

Open Comments:

Ascertainment of Exposure

Consensus Score: 1 — Report as 0 or 1

Reviewer 1 *Reviewer 2* *Conflicts*

AB	MK	MT	Value	Description
X	X	[redacted]	STAR	From a secure record (ex. Medical records)
		[redacted]	STAR	From a structured interview of a participant
		[redacted]	-	Written self-report by the participant
		[redacted]	-	No description of how the exposure was ascertained

Open Comments: AB - Prospectively collected database with good record of covariates

Demonstration that local recurrence had not already occurred at the start of the study

Consensus Score: 1 — Report as 0 or 1

Reviewer 1 *Reviewer 2* *Conflicts*

AB	MK	MT	Value	Description
X	X	[redacted]	STAR	Yes
		[redacted]	-	No

Open Comments:

COMPARABILITY

Comparability of cohorts on the basis of the design or analysis

Total Comparability Score: 1 — Out of **2** (note that the study can store two points in this single category)

Reviewer 1 *Reviewer 2* *Conflicts*

AB	MK	MT	Value	Description
	-	[redacted]	STAR	Study controls for tumour location
X	X	[redacted]	STAR	Study controls for chemotherapy (multi-agent; neoadjuvant and/or adjuvant)

Open Comments: (MJK) Table 1 shows similar location, but this was coincidence not by design

OUTCOME

Total Selection Score: 2 — Out of 3

Assessment of Outcome

Consensus Score: 1 — Report as 0 or 1

AB	MK	MT	Value	Description
X	X	[redacted]	STAR	Independent blind assessment (ex. Linked to medical records)
		[redacted]	STAR	Record linkage (ex. Automated database extraction, like billing codes)
		[redacted]	-	Self-reported by participant (i.e. no reference to the medical record)
		[redacted]	-	No description

Open Comments:

Was follow-up long-enough for outcomes to occur

Consensus Score: 1 — Report as 0 or 1

AB	MK	MT	Value	Description
X	X	[redacted]	STAR	Yes (Median FU: 3 years)
		[redacted]	-	No

Open Comments: Media F/U 78 months

Adequacy of Follow-up

Consensus Score: 0 — Report as 0 or 1

AB	MK	MT	Value	Description
		[redacted]	STAR	Complete follow-up - all subjects accounted for
		[redacted]	STAR	Subjects lost to follow-up unlikely to introduce bias - small number lost (85% have adeq FU)
		[redacted]	-	Follow-up rate was under 85% and no description for those lost to follow-up
X	X	[redacted]	-	No statement

Open Comments:

First Author: Kennecke **Total** **7**

SELECTION

Total Selection Score: 4 — Out of 4

Representativeness of the Exposed Cohort

Consensus Score: 1 — Report as 0 or 1

AB	MT	JF	Value	Description
			STAR	Truly representative of T3N0 rectal cancer patients in the community
	X	X	STAR	Somewhat representative of T3N0 rectal cancer patients in the community
X			-	Not well representing T3N0 rectal cancer patients in the community
			-	No description of the derivation of the cohort

Open Comments: AB - Had a hard time deciding this one, but erred on the side of bias as we don't know the % of TME
MT-there are non-TME patients, which may inflate LR
JF: It is somewhat representative: they somewhat included TME patients.

Selection of the Non-Exposed Cohort

Consensus Score: 1 — Report as 0 or 1

AB	MT	JF	Value	Description
	X	X	STAR	Drawn from the same community as the exposed cohort
X			-	Drawn from a different community than the exposed cohort
			-	No description of how the non-exposed cohort was derived

Open Comments: AB - Differences in age noted. Significant as age <70 for surgery only was 29% vs. 77% for ChemoRT group
MT- it's the same community though? just different pts make up?
JF: same community. Not sure if differences as baseline count as "drawn from a different community"

Ascertainment of Exposure

Consensus Score: 1 — Report as 0 or 1

Reviewer 1 *Reviewer 2* *Conflicts*

AB	MT	JF	Value	Description
X	X	[redacted]	STAR	From a secure record (ex. Medical records)
		[redacted]	STAR	From a structured interview of a participant
		[redacted]	-	Written self-report by the participant
		[redacted]	-	No description of how the exposure was ascertained

Open Comments:

Demonstration that local recurrence had not already occurred at the start of the study

Consensus Score: 1 — Report as 0 or 1

Reviewer 1 *Reviewer 2* *Conflicts*

AB	MT	JF	Value	Description
X	X	[redacted]	STAR	Yes
		[redacted]	-	No

Open Comments:

COMPARABILITY

Comparability of cohorts on the basis of the design or analysis

Total Comparability Score: 1 — Out of **2** (note that the study can store two points in this single category)

Reviewer 1	*Reviewer 2*	*Conflicts*		
**AB**	**MT**	**JF**	**Value**	**Description**
X	X		*STAR*	Study controls for tumour location
			STAR	Study controls for chemotherapy (multi-agent; neoadjuvant and/or adjuvant)

Open Comments: AB - documents some characteristics of chemo, but does not control

OUTCOME

Total Selection Score: 2 — Out of 3

Assessment of Outcome

Consensus Score: 1 — Report as 0 or 1

Reviewer 1	*Reviewer 2*	*Conflicts*		
**AB**	**MT**	**JF**	**Value**	**Description**
X	X		*STAR*	Independent blind assessment (ex. Linked to medical records)
			STAR	Record linkage (ex. Automated database extraction, like billing codes)
			-	Self-reported by participant (i.e. no reference to the medical record)
			-	No description

Open Comments:

Was follow-up long-enough for outcomes to occur

Consensus Score: 1 — Report as 0 or 1

Reviewer 1	*Reviewer 2*	*Conflicts*		
**AB**	**MT**	**JF**	**Value**	**Description**
X	X		*STAR*	Yes (Median FU: 3 years)
			-	No

Open Comments: AB - 5.2 years

Adequacy of Follow-up

Consensus Score: 0 — Report as 0 or 1

Reviewer 1	*Reviewer 2*	*Conflicts*		
**AB**	**MT**	**JF**	**Value**	**Description**
			STAR	Complete follow-up - all subjects accounted for
			STAR	Subjects lost to follow-up unlikely to introduce bias - small number lost (at least 85% have adequate follow-up and/or a description of why they were lost to follow-up)
			-	Follow-up rate was under 85% and no description for those lost to follow-up
X	X		-	No statement

Open Comments:

First Author: Wu **Total** 8

SELECTION

Total Selection Score: 4 — Out of 4

Representativeness of the Exposed Cohort

Consensus Score: 1 — Report as 0 or 1

Reviewer 1	*Reviewer 2*	*Conflicts*		
**JF**	**MK**	**AB**	**Value**	**Description**
X		X	*STAR*	Truly representative of T3N0 rectal cancer patients in the community
	X		*STAR*	Somewhat representative of T3N0 rectal cancer patients in the community
			-	Not well representing T3N0 rectal cancer patients in the community
			-	No description of the derivation of the cohort

Open Comments: JF: T3N0 patients, exposed to TME +- RT. Irrespective of resolution of conflict, it gets a star.
AB: Patients were skewed towards low rectal (0-5cm) at 50%, otherwise I think it's representative

Selection of the Non-Exposed Cohort

Consensus Score: 1 — Report as 0 or 1

Reviewer 1	*Reviewer 2*	*Conflicts*		
**JF**	**MK**	**AB**	**Value**	**Description**
X	X		*STAR*	Drawn from the same community as the exposed cohort
			-	Drawn from a different community than the exposed cohort
			-	No description of how the non-exposed cohort was derived

Open Comments: JF: Retrospective study, same Hospital.

Ascertainment of Exposure

Consensus Score: 1 — Report as 0 or 1

Reviewer 1	*Reviewer 2*	*Conflicts*		
**JF**	**MK**	**AB**	**Value**	**Description**
X	X		*STAR*	From a secure record (ex. Medical records)
			STAR	From a structured interview of a participant
			-	Written self-report by the participant
			-	No description of how the exposure was ascertained

Open Comments: JF: Medical records from hospital in China. 2003 - 2011 review.

Demonstration that local recurrence had not already occurred at the start of the study

Consensus Score: 1 — Report as 0 or 1

Reviewer 1	*Reviewer 2*	*Conflicts*		
**JF**	**MK**	**AB**	**Value**	**Description**
X			*STAR*	Yes
			-	No

Open Comments: JF: Mike, you missed marking this part.

COMPARABILITY

Comparability of cohorts on the basis of the design or analysis

Total Comparability Score: 2 — Out of **2** (note that the study can store two points in this single category)

Reviewer 1	*Reviewer 2*	*Conflicts*		
**JF**	**MK**	**AB**	**Value**	**Description**
X	X		*STAR*	Study controls for tumour location
X	X		*STAR*	Study controls for chemotherapy (multi-agent; neoadjuvant and/or adjuvant)

Open Comments: JF: Pateints received CRT on intervention arm vs chemotherapy alone in the control arm.

OUTCOME

Total Selection Score: 2 — Out of 3

Assessment of Outcome

Consensus Score: 1 — Report as 0 or 1

Reviewer 1	*Reviewer 2*	*Conflicts*		
**JF**	**MK**	**AB**	**Value**	**Description**
X	X		*STAR*	Independent blind assessment (ex. Linked to medical records)
			STAR	Record linkage (ex. Automated database extraction, like billing codes)
			-	Self-reported by participant (i.e. no reference to the medical record)
			-	No description

Open Comments: JF: outcome evaluated directly from medical records.

Was follow-up long-enough for outcomes to occur

Consensus Score: 1 — Report as 0 or 1

Reviewer 1	*Reviewer 2*	*Conflicts*		
**JF**	**MK**	**AB**	**Value**	**Description**
X	X		*STAR*	Yes (Median FU: 3 years)

			-	No

Open Comments: JF: self-explanatory.

Adequacy of Follow-up

Consensus Score: 0 — Report as 0 or 1

Reviewer 1 JF	*Reviewer 2* MK	*Conflicts* AB	Value	Description
			STAR	Complete follow-up - all subjects accounted for
			STAR	Subjects lost to follow-up unlikely to introduce bias - small number lost (at least 85% have adequate follow-up and/or a description of why they were lost to follow-up)
X	X		-	Follow-up rate was under 85% and no description for those lost to follow-up
			-	No statement

Open Comments:

First Author: Lin **Total** 8

SELECTION

Total Selection Score: 4 — Out of 4

Representativeness of the Exposed Cohort

Consensus Score: 1 — Report as 0 or 1

Reviewer 1 JF	*Reviewer 2* MT	*Conflicts* MK	Value	Description
			STAR	Truly representative of T3N0 rectal cancer patients in the community
X	X		*STAR*	Somewhat representative of T3N0 rectal cancer patients in the community
			-	Not well representing T3N0 rectal cancer patients in the community
			-	No description of the derivation of the cohort

Open Comments:

Selection of the Non-Exposed Cohort

Consensus Score: 1 — Report as 0 or 1

Reviewer 1 JF	*Reviewer 2* MT	*Conflicts* MK	Value	Description
X	X		*STAR*	Drawn from the same community as the exposed cohort
			-	Drawn from a different community than the exposed cohort
			-	No description of how the non-exposed cohort was derived

Open Comments:

Ascertainment of Exposure

Consensus Score: 1 — Report as 0 or 1

Reviewer 1 JF	*Reviewer 2* MT	*Conflicts* MK	Value	Description
X	X		*STAR*	From a secure record (ex. Medical records)
			STAR	From a structured interview of a participant
			-	Written self-report by the participant
			-	No description of how the exposure was ascertained

Open Comments:

Demonstration that local recurrence had not already occurred at the start of the study

Consensus Score: 1 — Report as 0 or 1

Reviewer 1 JF	*Reviewer 2* MT	*Conflicts* MK	Value	Description
	X		*STAR*	Yes
			-	No

Open Comments: MT: pre treatment imaging w MR n US done

COMPARABILITY

Comparability of cohorts on the basis of the design or analysis

Total Comparability Score: 2 — Out of **2** (note that the study can store two points in this single category)

Reviewer 1 JF	*Reviewer 2* MT	*Conflicts* MK	Value	Description
X	X		*STAR*	Study controls for tumour location
X	X		*STAR*	Study controls for chemotherapy (multi-agent; neoadjuvant and/or adjuvant)

Open Comments: yes for chemo as all neoadj

OUTCOME

Total Selection Score: 2 — Out of 3

Assessment of Outcome

Consensus Score: 1 — Report as 0 or 1

Reviewer 1 JF	*Reviewer 2* MT	*Conflicts* MK	Value	Description
X	X		*STAR*	Independent blind assessment (ex. Linked to medical records)
			STAR	Record linkage (ex. Automated database extraction, like billing codes)
			-	Self-reported by participant (i.e. no reference to the medical record)
			-	No description

Open Comments:

Was follow-up long-enough for outcomes to occur

Consensus Score: 1 — Report as 0 or 1

Reviewer 1 JF	*Reviewer 2* MT	*Conflicts* MK	Value	Description
X	X		*STAR*	Yes (Median FU: 3 years)
			-	No

Open Comments:

Adequacy of Follow-up

Consensus Score: 0 — Report as 0 or 1

Reviewer 1 JF	*Reviewer 2* MT	*Conflicts* MK	Value	Description
			STAR	Complete follow-up - all subjects accounted for
	X		*STAR*	Subjects lost to follow-up unlikely to introduce bias - small number lost (at least 85% have adequate follow-up and/or a description of why they were lost to follow-up)
			-	Follow-up rate was under 85% and no description for those lost to follow-up
X		X	-	No statement

Open Comments: JF: I could not find any statement as to the adequacy of follow up.
MK - I see median FU stated (which is addressed in the prior evaluation), but no comments regarding FUs adequacy

First Author: Peng **Total** 9

SELECTION

Total Selection Score: 4 — Out of 4

Representativeness of the Exposed Cohort

Consensus Score: 1 — Report as 0 or 1

Reviewer 1 MK	*Reviewer 2* MT	*Conflicts* AB	Value	Description
			STAR	Truly representative of T3N0 rectal cancer patients in the community
X	X		*STAR*	Somewhat representative of T3N0 rectal cancer patients in the community
			-	Not well representing T3N0 rectal cancer patients in the community

			-	No description of the derivation of the cohort

Open Comments:

Selection of the Non-Exposed Cohort

Consensus Score: 1 Report as 0 or 1

Reviewer 1 MK	*Reviewer 2* MT	*Conflicts* AB	Value	Description
X	X		*STAR*	Drawn from the same community as the exposed cohort
			-	Drawn from a different community than the exposed cohort
			-	No description of how the non-exposed cohort was derived

Open Comments:

Ascertainment of Exposure

Consensus Score: 1 Report as 0 or 1

Reviewer 1 MK	*Reviewer 2* MT	*Conflicts* AB	Value	Description
X	X		*STAR*	From a secure record (ex. Medical records)
			STAR	From a structured interview of a participant
			-	Written self-report by the participant
			-	No description of how the exposure was ascertained

Open Comments:

Demonstration that local recurrence had not already occurred at the start of the study

Consensus Score: 1 Report as 0 or 1

Reviewer 1 MK	*Reviewer 2* MT	*Conflicts* AB	Value	Description
X	X		*STAR*	Yes
			-	No

Open Comments:

COMPARABILITY

Comparability of cohorts on the basis of the design or analysis

Total Comparability Score: 2 Out of **2** (note that the study can store two points in this single category)

Reviewer 1 MK	*Reviewer 2* MT	*Conflicts* AB	Value	Description
X	X		*STAR*	Study controls for tumour location
X	X		*STAR*	Study controls for chemotherapy (multi-agent; neoadjuvant and/or adjuvant)

Open Comments:

OUTCOME

Total Selection Score: 3 Out of 3

Assessment of Outcome

Consensus Score: 1 Report as 0 or 1

Reviewer 1 MK	*Reviewer 2* MT	*Conflicts* AB	Value	Description
X	X		*STAR*	Independent blind assessment (ex. Linked to medical records)
			STAR	Record linkage (ex. Automated database extraction, like billing codes)
			-	Self-reported by participant (i.e. no reference to the medical record)
			-	No description

Open Comments:

Was follow-up long-enough for outcomes to occur

Consensus Score: 1 Report as 0 or 1

Reviewer 1 MK	*Reviewer 2* MT	*Conflicts* AB	Value	Description
X	X		*STAR*	Yes (Median FU: 3 years)
			-	No

Open Comments:

Adequacy of Follow-up

Consensus Score: 1 Report as 0 or 1

Reviewer 1 MK	*Reviewer 2* MT	*Conflicts* AB	Value	Description
			STAR	Complete follow-up - all subjects accounted for
X	X		*STAR*	Subjects lost to follow-up unlikely to introduce bias - small number lost (at least 85% have adequate follow-up and/or a description of why they were lost to follow-up)
			-	Follow-up rate was under 85% and no description for those lost to follow-up
			-	No statement

Open Comments:

First Author: Baek **Total** 8

SELECTION

Total Selection Score: 4 Out of 4

Representativeness of the Exposed Cohort

Consensus Score: 1 Report as 0 or 1

Reviewer 1 MK	*Reviewer 2* MT	*Conflicts* JF	Value	Description
			STAR	Truly representative of T3N0 rectal cancer patients in the community
X	X		*STAR*	Somewhat representative of T3N0 rectal cancer patients in the community
			-	Not well representing T3N0 rectal cancer patients in the community
			-	No description of the derivation of the cohort

Open Comments:

Selection of the Non-Exposed Cohort

Consensus Score: 1 Report as 0 or 1

Reviewer 1 MK	*Reviewer 2* MT	*Conflicts* JF	Value	Description
X	X		*STAR*	Drawn from the same community as the exposed cohort
			-	Drawn from a different community than the exposed cohort
			-	No description of how the non-exposed cohort was derived

Open Comments:

Ascertainment of Exposure

Consensus Score: 1 Report as 0 or 1

Reviewer 1 MK	*Reviewer 2* MT	*Conflicts* JF	Value	Description
X	X		*STAR*	From a secure record (ex. Medical records)
			STAR	From a structured interview of a participant
			-	Written self-report by the participant
			-	No description of how the exposure was ascertained

Open Comments:

Demonstration that local recurrence had not already occurred at the start of the study

Consensus Score: 1 Report as 0 or 1

Reviewer 1 MK	*Reviewer 2* MT	*Conflicts* JF	Value	Description

X	X		*STAR*	Yes
			-	No

Open Comments:

COMPARABILITY

Comparability of cohorts on the basis of the design or analysis

Total Comparability Score: 2 — Out of **2** (note that the study can store two points in this single category)

Reviewer 1 MK	*Reviewer 2* MT	*Conflicts* JF	Value	Description
X	X		*STAR*	Study controls for tumour location
X	X		*STAR*	Study controls for chemotherapy (multi-agent; neoadjuvant and/or adjuvant)

Open Comments:

OUTCOME

Total Selection Score: 2 — Out of 3

Assessment of Outcome

Consensus Score: 1 — Report as 0 or 1

Reviewer 1 MK	*Reviewer 2* MT	*Conflicts* JF	Value	Description
X	X		*STAR*	Independent blind assessment (ex. Linked to medical records)
			STAR	Record linkage (ex. Automated database extraction, like billing codes)
			-	Self-reported by participant (i.e. no reference to the medical record)
			-	No description

Open Comments:

Was follow-up long-enough for outcomes to occur

Consensus Score: 1 — Report as 0 or 1

Reviewer 1 MK	*Reviewer 2* MT	*Conflicts* JF	Value	Description
X	X		*STAR*	Yes (Median FU: 3 years)
			-	No

Open Comments:

Adequacy of Follow-up

Consensus Score: 0 — Report as 0 or 1

Reviewer 1 MK	*Reviewer 2* MT	*Conflicts* JF	Value	Description
			STAR	Complete follow-up - all subjects accounted for
	X		*STAR*	Subjects lost to follow-up unlikely to introduce bias - small number lost (at least 85% have adequate follow-up and/or a description of why they were lost to follow-up)
			-	Follow-up rate was under 85% and no description for those lost to follow-up
X		X	-	No statement

Open Comments: MT: they hv statement in Methods under "patients", and median follow up of 71 months, likely by 24 months they still have most of their peeps (looking at the Kms, not that many losses upfront).
MJK: following review, I still don't see a statement regarding loss to to FU. I feel that median FU is different. For example, the patient that was followed for only 3 mths, was that because of lost to FU or were they just added to the database?
JF: I did not find an statement referring to the "adequacy of follow up". In retrospective studies this is seldom reported, I think. Different form randomized trials. Anyways, on this study, we just do not have a compelling statement, either in the methods or patients section.
